# Trends in the Range of Zero Alcohol Products Available in Supermarkets and Alcohol Stores in Australia

**DOI:** 10.1111/dar.70122

**Published:** 2026-03-03

**Authors:** Simone Pettigrew, Tazman Davies, Bella Sträuli, Asad Yusoff, Paula O'Brien, Michelle Jongenelis, Aimee Brownbill, Alexandra Jones, Fraser Taylor, Jacquie Bowen

**Affiliations:** ^1^ The George Institute for Global Health, UNSW Sydney Sydney Australia; ^2^ School of Medicine and Dentistry Griffith University Gold Coast Australia; ^3^ University of Melbourne Melbourne Australia; ^4^ Melbourne Centre for Behaviour Change, Melbourne School of Psychological Sciences The University of Melbourne Melbourne Australia; ^5^ Foundation for Alcohol Research and Education Canberra Australia; ^6^ National Centre for Education and Training on Addiction, Flinders Health and Medical Research Institute Flinders University Adelaide Australia

**Keywords:** brands, children, retail, supermarkets, zero alcohol products

## Abstract

**Introduction:**

With some exceptions, alcohol cannot be sold in Australian supermarkets. The emerging availability of zero alcohol products (ZAP) in supermarkets is therefore introducing alcohol brands into this previously protected domain. The aims of the present study were to examine the prevalence of ZAPs in supermarkets, compare this to the prevalence of ZAPs in alcohol stores, and assess the extent to which ZAPs available in supermarkets share branding with alcohol products.

**Methods:**

ZAPs available for sale in large supermarket and alcohol chain stores in Australia were assessed in 2022 and 2024. Analyses examined: (i) ZAPs available in each store type; (ii) alcohol brand extension ZAPs available in supermarkets; (iii) overlap in ZAP brands between supermarkets and alcohol stores; and (iv) changes in (i)–(iii) over time.

**Results:**

The number of unique ZAPs available for sale in supermarkets remained roughly stable between 2022 (*n* = 70) and 2024 (*n* = 66). A substantial increase in ZAPs prevalence was observed in alcohol stores (*n* = 110 in 2022 to *n* = 261 in 2024). By 2024, ZAPs with alcohol brands dominated supermarkets, representing 59% of available ZAPs, up from 37% in 2022. The percentage of unique ZAPs that were available in both supermarkets and alcohol stores remained stable over the period at 18%.

**Discussion and Conclusions:**

The new dominance of alcohol branded ZAPs in supermarkets demonstrates how alcohol branding is increasingly permeating the supermarket environment in Australia. The resulting increased exposure of children and adolescents to alcohol‐related stimuli should be considered in ZAPs policies to minimise adverse outcomes.

## 1 Introduction

The emerging market of zero alcohol products (ZAP) brings a range of potential positive and negative outcomes. In terms of possible positive effects, consumers may choose to substitute alcoholic beverages with ZAP equivalents to reduce their alcohol intake [1], which in turn could reduce alcohol‐related harms for individuals and communities [2]. Definitive evidence of this effect is lacking [3], likely due to the relative recency of the evolution of this product category [4, 5, 6]. However, there is evidence from the UK that households may be replacing some of their usual alcohol intake with ZAPs [7], and those with higher‐risk alcohol use may be using ZAPs to assist them in reducing their alcohol intake [1, 8]. Potential negative outcomes from ZAPs include the possibility that these products are additions rather than substitutes in individuals' overall drinking patterns, which the World Health Organization notes has the potential to reinforce an alcohol culture [6]. Other noted issues include the triggering effects of exposure to alcohol‐related stimuli in a broader range of contexts [9], especially for youth and individuals with alcohol dependency [10], and the possible gateway effect from young people consuming the products and becoming acclimatised to alcohol flavours from a younger age [4, 11].

In Australia, the context of the present study, alcoholic beverages are typically not permitted to be sold in supermarkets [12], which has effectively ensured these stores have remained free of alcohol‐related advertising material. The advent of ZAPs has therefore presented the opportunity for alcohol companies to bring their brands into this previously protected domain. This situation is likely to be of considerable value to these companies due to the role of brands as unique product identifiers that facilitate recall and recognition to promote purchasing [13], and the role of brand equity (i.e., the financial value of the brand) in contributing to a company's net worth [14]. There are no legal restrictions on selling ZAPs to underage drinkers in Australia, although some retailers are voluntarily choosing not to do so [15]. Concerns have been expressed about the typical location of ZAPs in soft‐drink aisles of supermarkets where they are likely to be seen by children [11, 16]. Exposure among youth appears to be substantial, with Australian research finding that more than half of 14–17 year olds recalled seeing ZAPs in supermarkets and more than one‐third had tried at least one type of ZAP [11].

The growing availability in supermarkets of ZAPs that share alcohol product branding constitutes a form of stealth marketing that adds to the overall level of alcohol brand exposure [17, 18]. It can also be considered as a form of integrated marketing communication, which involves promoting brands across a range of channels to optimise consumer awareness of the products and stimulate purchase intentions [19]. This approach relies on the ‘mere exposure effect’ that results in subconscious coding of brand information to memory [20]. This can occur regardless of whether the individual is aware of the exposure [21, 22, 23] and can manifest in strengthened product preference over time [22, 23, 24]. The subconscious nature of this process can prevent individuals from being able to form mental counter‐arguments to protect themselves from developing positive attitudes to the promoted products [24]. Incidental exposures to alcohol‐related stimuli can influence attitudes to both specific brands and the entire product category [25].

Of particular concern are the potential effects of alcohol brand exposure in supermarkets on youth [11]. Reaction time research undertaken with adolescents suggests ZAPs (defined in the study as < 0.5% alcohol by volume) are often mentally coded as alcohol products [26]. Greater exposure to alcohol‐related stimuli across a range of contexts has been found to increase the risk of early alcohol initiation and the amount consumed [25, 27, 28, 29]. Illustrating the power of integrated marketing communications, previous research has found that adolescents are at greater risk of early alcohol use when they are exposed to alcohol marketing across multiple channels [28], including in retail stores where alcohol is sold [30, 31, 32].

In combination, these bodies of evidence suggest that additional exposures to alcohol‐related stimuli resulting from the introduction of alcohol brands in supermarkets could increase the risk of alcohol‐related harm among young people [11]. It is therefore important to monitor the extent to which ZAPs are penetrating supermarket environments and whether the ZAPs that are available share brand names with existing alcohol products, thereby providing a source of brand promotion in a new context that is frequented by people of all ages. Accordingly, the aims of the present study were to examine the availability of ZAPs in supermarkets, compare this to the prevalence of ZAPs in alcohol stores, identify the extent of overlap between ZAPs sold in both supermarkets and alcohol stores, and assess the extent to which ZAPs available in supermarkets share branding with alcohol products. In addition, changes in these outcomes were assessed between 2022 and 2024 to identify any trends over time.

## 2 Methods

This study used data from the FoodSwitch database and the Alcohol Industry Monitoring System (AIMS) database, both of which are compiled and managed by The George Institute for Global Health. Trained data collectors annually visit a selection of supermarkets and alcohol stores that represent the major food (Coles, Woolworths, IGA, Aldi, Harris Farm) and alcohol (Dan Murphy's, BWS and Liquorland) retail chains and take photographs of all sides of products available for sale. From 2024 onwards, some of the stores' websites were scraped to obtain product data rather than data collectors visiting the stores in‐person due to company permission to collect data on‐site being withdrawn. All visited and web‐scraped stores are located in Sydney, Australia. Trained coders extract information from the in‐store photographs and web‐scraped images to generate comprehensive datasets of food and beverage product attributes (e.g., product name, beverage type and percent alcohol by volume). Coders independently enter attributes from the product label images, with checks and validation undertaken by a second coder. In addition, automated checking processes are used to identify anomalies (e.g., whether the ABV% of each product aligns with the norms observed in the relevant alcohol product category).

For the present study, beverage data from supermarkets and alcohol stores from the 2022 and 2024 collections were accessed to obtain insights into trends in ZAPs availability in recent years. For the in‐store visits, supermarket data were collected March–August in 2022 and April–November in 2024. Alcohol store data were collected March–May in 2022 and May–June in 2024. The web‐scraped data were captured in September 2024. Products were classified as ZAPs if they were described as alcohol variants (e.g., wine, beer, spirits, cider, premix) and the alcohol content was ≤ 0.5% of the total volume. Product bar codes were used to identify unique products and to match products sold in both supermarkets and alcohol outlets. Pack size variants of the same product were counted as two separate products, reflecting the additional shelf exposure obtained through multiple versions of the same product. Manual checks for all ZAPs were conducted online to determine whether they were brand extensions of alcohol brands (e.g., Guinness Draught and Guinness Draught 0.0).

The proportion of ZAPs was calculated in both supermarkets and alcohol stores as the percentage of the total number of unique beverages available, including soft drinks, waters, juices, cordials and mixers (i.e., excluding dairy beverages). The prevalence of ZAPs in supermarkets and alcohol stores was assessed overall and by alcohol category (wine, beer, spirits, premix, cider and other). Pearson chi‐square (*χ* ^2^) tests were used to assess whether the prevalence of ZAPs and alcohol brand extensions differed between 2022 and 2024, where a *p*‐value less than 0.05 was considered statistically significant.

## 3 Results

In both 2022 (*n* = 70, 2.3%) and 2024 (*n* = 66, 2.2%), ZAPs represented small proportions of the total number of beverages available for sale in supermarkets (see Table 1). There was some change in distribution by ZAP product category, with the number of unique beer products increasing (from 20 to 27) while the number of spirits (7 to 3) and premix (18 to 11) decreased. However, none of these differences were statistically significant.

**TABLE 1:** Prevalence of unique ZAPs in major supermarket chain stores, as a proportion of all beverages.

	2022		2024	
	Total, *n*	ZAPs, *n* (%^a^)	Total, *n*	ZAPs, *n* (%^a^)
All categories	3003	70 (2.3)	3022	66 (2.2)
Wine	24	24 (100.0)	25	25 (100.0)
Spirits	7	7 (100.0)	3	3 (100.0)
Beer	20	20 (100.0)	27	27 (100.0)
Premix	18	18 (100.0)	11	11 (100.0)
Cider	0	—	0	—
Other^b^	1	1 (100.0)	0	—
Soft drinks, waters, juices, cordials, mixers	2933	0 (0.0)	2956	0 (0.0)

*Note:* No significant differences in prevalence between years.

Abbreviation: ZAPs, zero alcohol products.

^a^ Percentage of all beverages available for sale.

^b^ Sake product.

Somewhat different trends were observed for ZAPs sold in the sampled alcohol stores (see Table 2). There was substantial and statistically significant growth in the number of unique ZAPs from *n* = 110 in 2022 to *n* = 261 in 2024. Most of this increase was driven by growth in the wine, beer and spirits ZAPs categories. However, ZAPs remained a small proportion of the total number of beverages available for sale in alcohol stores each year, albeit with a 65% increase from 1.7% to 2.8%.

**TABLE 2:** Prevalence of unique ZAPs in major alcohol chain stores in 2022 and 2024, as a proportion of all beverages.

	2022		2024	
	All beverages, *n*	ZAPs, *n* (%^a^)	All beverages, *n*	ZAPs, *n* (%^a^)
All categories	6427	110 (1.7)	9454	261 (2.8^c^)
Wine	3356	43 (1.3)	5057	112 (2.2^c^)
Spirits	1085	12 (1.1)	1864	31 (1.7)
Beer	1185	48 (4.1)	1399	106 (7.6^c^)
Premix	468	6 (1.3)	859	7 (0.8)
Cider	179	1 (0.6)	129	4 (3.1)
Other^b^	65	0 (0.0)	24	1 (4.2)
Soft drinks, waters, juices, cordials, mixers	89	0 (0.0)	122	0 (0.0)

Abbreviation: ZAP, zero alcohol product.

^a^ Percentage of all beverages available for sale.

^b^ Mainly sake and soju products.

^c^ Change in prevalence statistically significant (*χ* ^2^ test, *p* < 0.05).

Reflecting the combination of the stable number of ZAPs in supermarkets and the substantial increase in ZAPs available in alcohol stores, the total number of unique ZAPs sold across both store types (i.e., in supermarkets and/or alcohol stores) increased from 152 in 2022 to 277 in 2024 (see Table 3). Despite this increase, the proportions of unique ZAPs that were sold in both types of stores remained stable over time (18.4% (28/152) in 2022 versus 18.1% (50/277) in 2024). None of the changes in total prevalence across store types between 2022 and 2024 were statistically significant.

**TABLE 3:** Prevalence of unique ZAPs available in both supermarkets and alcohol stores.

	2022		2024	
	All ZAPs^a^, *n*	ZAPs in both supermarket & alcohol stores, *n* (%)	All ZAPs^a^, *n*	ZAPs in both supermarket & alcohol stores, *n* (%)
All categories	152	28 (18.4)	277	50 (18.1)
Wine	54	13 (24.1)	115	22 (19.1)
Spirits	16	3 (18.8)	31	3 (9.7)
Beer	57	11 (19.3)	111	22 (19.8)
Premix	23	1 (4.3)	15	3 (20.0)
Cider	1	0 (0.0)	4	0 (0.0)
Other^b^	1	0 (0.0)	1	0 (0.0)

*Note:* No significant differences in prevalence between years.

Abbreviation: ZAPs, zero alcohol products.

^a^ Total of unique ZAP products available in sampled supermarket and alcohol stores.

^b^ Mainly sake and soju products.

Table 4 shows the growth in the number of ZAPs constituting alcohol brand extensions that were sold in the sampled supermarkets. In 2022, 37.1% of ZAPs sold in supermarkets shared alcohol brand names, significantly increasing to 59.1% in 2024. Beer products accounted for most of this increase; by 2024, 85.2% of beer ZAPs available in the supermarkets were brand extensions of alcohol products compared to 65.0% in 2022.

**TABLE 4:** Prevalence of alcohol brand extension ZAPs in supermarkets.

	2022		2024	
	Total ZAPs in supermarkets, *n*	ZAPs with an alcohol brand extension in supermarkets, *n* (%)	Total ZAPs in supermarkets, *n*	ZAPs with an alcohol brand extension in supermarkets, *n* (%)
All categories	70	26 (37.1)	66	39 (59.1^b^)
Wine	24	11 (45.8)	25	13 (52.0)
Spirits	7	1 (14.3)	3	1 (33.3)
Beer	20	13 (65.0)	27	23 (85.2)
Premix	18	1 (5.6)	11	2 (18.2)
Cider	0	—	0	—
Other^a^	1	0 (0.0)	0	—

Abbreviation: ZAP, zero alcohol product.

^a^ Mainly sake and soju products.

^b^ Change in prevalence statistically significant (*χ* ^2^ test, *p* < 0.05).

## 4 Discussion

The study results indicate a small presence of ZAPs in supermarkets and alcohol stores relative to the total number of beverages available. The proportions remained roughly stable in supermarkets between 2022 and 2024; however, there was a 65% increase in availability in the sampled alcohol stores, indicating a rapid rate of growth for the product category within this retailer type. This outcome is aligned with industry analysis reports indicating increasing market penetration of zero and low alcohol products [33, 34]. The findings are also consistent with UK research demonstrating substantial growth in the availability of ZAPs, but that they only account for a small proportion of total consumption of alcoholic and zero alcohol products combined [7].

A key finding was the apparent consolidation across supermarket and alcohol store contexts in terms of the ZAPs available in supermarkets increasingly representing brand extensions of alcohol products. This is a tangible representation of integrated marketing, which involves consumers being exposed to the same branding in multiple locations to optimise awareness, liking and preference [19, 21], in this case likely resulting in increased exposure of supermarket visitors, including children and youth, to established alcohol brands. As per the ‘mere exposure effect’ [20, 22, 23, 24], shared branding across supermarket and alcohol stores can be expected to increase brand familiarity and potentially result in earlier use of the alcoholic versions of the promoted brands due to increased brand awareness. Previous Australian research with adolescents found many recalled seeing ZAPs in supermarkets, with some expressing concern about the possible gateway effects of these products, resulting in recommendations from some of the sampled adolescents that ZAPs be placed in low‐visibility areas of supermarkets (e.g., in a back corner) or removed from these outlets altogether [11].

The growing presence of alcohol‐branded ZAPs in supermarkets also warrants consideration in light of evidence from e‐cigarette retailing demonstrating that exposure to point‐of‐sale advertising contributes to youth uptake of both e‐cigarettes and tobacco products [35]. Similarly, ZAPs' integration into everyday shopping contexts may reinforce alcohol brand salience and facilitate gateway effects, particularly among adolescents who mentally code these products as alcohol‐related [26]. These parallels suggest that recommended regulatory responses developed for e‐cigarettes, such as restrictions on in‐store placement and display bans [36], could be considered for ZAPs to mitigate unintended consequences.

In the absence to date of definitive evidence of positive benefits of ZAPs, it is imperative that any potential benefits are weighed against the effects of alcohol brand advertising becoming even more ubiquitous. A dose–response relationship has been identified whereby incremental exposure to alcohol marketing among underage youth is associated with increased consumption of the promoted brands [37]. Additional brand exposures in supermarket environments therefore matter and research is needed to quantify the effects [15], although it is important to also acknowledge the extensive array of other ways in which young people are likely to be exposed to ZAPs advertising, including through social media influencers and sports sponsorship [9, 38]. To date there is little evidence of the extent to which this exposure is influencing youth [39], but a precautionary approach is deemed to be warranted [6, 40].

The primary limitation of this study was the use of data collected from stores in a single city, Sydney. Consequently, the results cannot be assumed to reflect the availability of ZAPs in supermarkets and alcohol stores across the country. Second, audits of physical and online stores only provide access to availability data relevant to retail contexts, and there are various other contexts in which individuals are exposed to both alcohol and zero‐alcohol products (e.g., restaurants, bars, sporting events). Third, the need to use web‐scraping to collect some of the 2024 data could have confounded the results through differences in ranges sold online versus in‐store at the time of data collection, including the potential for products to have been included in the later dataset that are only ever available for sale online, reducing comparability. However, identical coding and analysis processes were used for the in‐store and web‐scraped product images, minimising any impacts of the data collection method change for the 2024 data. As further annual collections are added to the AIMS database, it will be possible to conduct detailed analyses of the extent to which the web‐scraped data differ from data collected in‐store.

Fourth, this study analysed data from just two time points. Future studies could assess the prominence of zero alcohol brands in a broader range of provision contexts and utilise data from a wider geographical area over a longer time period to better identify relevant trends in the availability of ZAPs. Finally, the product availability data examined in this study are unable to provide insights into consumers' responses when exposed to alcohol branding in the form of ZAPs in varying types of outlets. This is a key area of further study because the results will inform the ways in which policymakers should regulate ZAPs advertising and other forms of ZAPs promotion (e.g., sports sponsorship) to reduce any potential adverse outcomes. Future research should attempt to unpack the differential effects of ZAPs marketing exposure in varying contexts, including in terms of whether different population sub‐groups react in different ways. In particular, it is important to investigate whether children and adolescents have different conscious and subconscious responses to ZAPs given their greater susceptibility to substance‐related stimuli compared to adults who have more fully developed cognitive abilities [41].

In conclusion, the results of this study highlight the importance of monitoring the growth in the ZAPs market across different types of retail outlets to assess the extent to which these products are available for those wanting to reduce their alcohol intake, the extent to which alcohol brands are dominating the ZAPs market, and the implications for youth exposure to alcohol‐related stimuli. How and where young people are being exposed to alcohol branding in new locations should be considered in the development of policies on the availability and promotion of ZAPs.

## Author Contributions

Conceptualisation: S.P. Methodology: S.P. Formal analysis: T.D. Investigation: S.P. B.S., A.Y. Writing – original draft: S.P, T.D. Writing – Review and editing: B.S., A.Y., P.O'., M.J., A.B., A.J., F.T., J.B. Funding acquisition: S.P., P.O'., M.J., A.B., A.J., F.T., J.B.

## Funding

This study was funded by National Health and Medical Research Council (NHMRC) Ideas Grant APP2021186. S.P. is funded by NHMRC Investigator Grant #2034602.

## Conflicts of Interest

The authors declare no conflicts of interest.

## Data Availability

The data that support the findings of this study are available on request from the corresponding author. The data are not publicly available due to privacy or ethical restrictions.
